# Symptomatic post COVID patients have impaired alveolar capillary membrane function and high VE/VCO_2_

**DOI:** 10.1186/s12931-023-02602-3

**Published:** 2024-02-08

**Authors:** Piergiuseppe Agostoni, Massimo Mapelli, Elisabetta Salvioni, Irene Mattavelli, Cristina Banfi, Alice Bonomi, Maria Luisa Biondi, Sara Rovai, Gloria Tamborini, Manuela Muratori, Sarah Ghulam Ali, Stefania Ghilardi, Fabiana De Martino, Carlo Vignati, Pietro Palermo, Paola Gugliandolo, Davide Elia, Federica Moscucci, Roberto Cassandro, Daniele Andreini, Elisabetta Mancini, Sergio Harari

**Affiliations:** 1 Centro Cardiologico Monzino, IRCCS, Milan, Italy; 2 Department of Clinical Sciences and Community Health, University of Milan, Via Parea, 4, 20138 Milan, Italy; 3 Unità Funzionale di Cardiologia, Casa di Cura Tortorella, Salerno, Italy; 4 U.O. di Pneumologia e Terapia Semi-Intensiva Respiratoria, MultiMedica IRCCS, Milan, Italy; 5 DAI Internal Medicine and Medical Specialities, Policlinico Umberto I, Rome, Italy; 6 Division of University Cardiology, IRCCS Ospedale Galeazzi Sant’Ambrogio, Milan, Italy; 7 Department of Biomedical and Clinical Sciences, University of Milan, Milan, Italy

**Keywords:** Covid-19, Post COVID, Post-COVID-19 syndrome, Cardiopulmonary exercise test, Lung diffusion

## Abstract

**Background:**

Post COVID-19 syndrome is characterized by several cardiorespiratory symptoms but the origin of patients’ reported symptomatology is still unclear.

**Methods:**

Consecutive post COVID-19 patients were included. Patients underwent full clinical evaluation, symptoms dedicated questionnaires, blood tests, echocardiography, thoracic computer tomography (CT), spirometry including alveolar capillary membrane diffusion (DM) and capillary volume (Vcap) assessment by combined carbon dioxide and nitric oxide lung diffusion (DLCO/DLNO) and cardiopulmonary exercise test. We measured surfactant derive protein B (immature form) as blood marker of alveolar cell function.

**Results:**

We evaluated 204 consecutive post COVID-19 patients (56.5 ± 14.5 years, 89 females) 171 ± 85 days after the end of acute COVID-19 infection. We measured: forced expiratory volume (FEV_1_) 99 ± 17%pred, FVC 99 ± 17%pred, DLCO 82 ± 19%, DM 47.6 ± 14.8 mL/min/mmHg, Vcap 59 ± 17 mL, residual parenchymal damage at CT 7.2 ± 3.2% of lung tissue, peakVO_2_ 84 ± 18%pred, VE/VCO_2_ slope 112 [102–123]%pred. Major reported symptoms were: dyspnea 45% of cases, tiredness 60% and fatigability 77%. Low FEV_1_, Vcap and high VE/VCO_2_ slope were associated with persistence of dyspnea. Tiredness was associated with high VE/VCO_2_ slope and low PeakVO_2_ and FEV_1_ while fatigability with high VE/VCO_2_ slope. SPB was fivefold higher in post COVID-19 than in normal subjects, but not associated to any of the referred symptoms. SPB was negatively associated to Vcap.

**Conclusions:**

In patients with post COVID-19, cardiorespiratory symptoms are linked to VE/VCO_2_ slope. In these patients the alveolar cells are dysregulated as shown by the very high SPB. The Vcap is low likely due to post COVID-19 pulmonary endothelial/vasculature damage but DLCO is only minimally impaired being DM preserved.

**Supplementary Information:**

The online version contains supplementary material available at 10.1186/s12931-023-02602-3.

## Introduction

Many patients who recovered from SARS CoV-2 infection present a variety of symptoms which limits overall quality of life. Among those, reduced exercise and daily activities performance, dysfunctional breathing with and without hyperventilation, cough, dyspnea, weakness, and anxiety are frequently reported even after complete clinical COVID-19 resolution [1–5]. Of note, infection severity does not seem to play a role in the frequency and intensity of post COVID-19 symptoms [6, 7]. This condition has been named the post COVID-19 post COVID [8].

Several previous reports investigated the post-COVID-19 syndrome but conclusive results to explain the origin of symptoms are lacking [9]. Indeed, SARS CoV-2 infection, on top of the pulmonary damage, is characterized by a diffuse coagulopathy and vasculopathy [10–12], cardiac abnormalities as shown by elevated troponin level in several patients [13, 14], as well as neural, muscular, renal, and gastrointestinal dysfunction. To try to spread some light on this topic, we have undertaken the present research protocol applying a holistic cardio-respiratory approach.

## Methods

All consecutive patients referred to our cardiopulmonary laboratory with post-COVID-19 symptoms were evaluated but only subjects with COVID 19 related pneumonia were analyzed. Patients with severe diseases before COVID-19 infection were excluded from the study as were excluded patients with any disease which could per se influence exercise performance including heart failure, COPD, severe systemic hypertension, arrhythmias, cardiac ischemia, valvular hear disease or pulmonary hypertension. Differently, patients with pre COVID not-severe cardiorespiratory disease with mild and non-specific symptoms not affecting exercise performance were included in the study. All patients underwent a full clinical evaluation including a complete interview, laboratory tests, echocardiography (TTE), thoracic computer tomography (CT), cardiopulmonary exercise test (CPET) and spirometry with alveolar capillary membrane diffusion by means of combined carbon monoxide and nitric oxide lung diffusion (DLCO/DLNO). Since November 2020, in conjunction with the growing evidence of prolonged symptoms over time [15], we have systematically administered a dedicated questionnaire allowing to evaluate post COVID-19 symptoms including presence/absence of dyspnea either at rest or during activities, cough, tiredness, chest pain, joint pain, heart palpitation, diarrhea, anosmia, dysgeusia, exercise-induced fatigability and hair loss. The questionnaires were drawn-up by patients in a private room without external help, collected in a sealed envelope and analyzed only at the end of the entire study, to keep the healthcare personnel blinded.

Blood chemistry and complete arterial blood gas analysis were performed. As regards blood markers of alveolar capillary function we measured surfactant derived protein B (immature form; SPB) as previously described in details [16].

### Spirometry

Standard spirometry was performed at rest according to the American Thoracic Society and the European Respiratory Society criteria [17, 18]. Lung diffusion for carbon monoxide (DLCO) and nitric oxide (DLNO) were simultaneously measured in the standard sitting position through the single-breath technique, with a breath-hold time of 4 s (MS-PFT analyser, Jaeger Masterscreen, Hoechberg, Germany). Membrane diffusion (DM) subcomponent was calculated dividing DLNO by 1.97, while capillary volume (Vcap) was estimated = 1/θCO x [1/(1/DLCO – 1.97/DLNO) with 1/θCO = (0.73 + 0.0058 × alveolar PO_2_) × 14.6/Hb. We used as reference equations for DLCO and DM those proposed in official ERS technical standards [19, 20]. Differently for Vcap we utilized an up-to-date technical considerations and reference equations [22]. Alveolar volume (VA) was measured by helium decay slope [21].

### Transthoracic echocardiography.

TTE was performed using a Philips ultrasound machine (Epiq CVx-Philips Medical Systems, Andover, MA, USA). Complete standard bidimensional TTE analysis was accomplished. Left and right heart chambers volumes, systolic and diastolic function and systolic pulmonary artery pressure (sPAP) were obtained as previously described [23, 24]. The mitral and tricuspid valvular regurgitation grade was assessed by integrating semi-quantitative and quantitative methods [25].

### Thoracic computer tomography

CT examinations were performed using a 256-slices HRCT scanner (Revolution CT; GE Healthcare, Milwaukee, WI). No contrast media were administered to the patients. The percentage of extension of lung parenchyma affected by COVID-19 pneumonia was processed by a dedicated workstation (ADW4.6, GE Healthcare) using a specific reconstruction software (Thoracic-V-Car software; GE Healthcare). This quantitative approach enables an automated assessment of the pulmonary infection, depicting infection areas as high attenuation areas (HAAs) in respect of a defined threshold value ranging from 650 Hounsfield unit (HU) to 3071 HU. The amount of infected lung defined as the percentage of lung parenchyma above the predefined vendor-specific threshold of 650 HU (HAA%, HAA/total lung volume) was automatically calculated by the dedicated software for both lungs [26, 27].

### Cardiopulmonary exercise test

All CPETs were performed by means of a stationary ergospirometer (Quark PFT Cosmed, Rome, Italy) using an electronically braked cycle ergometer. The progressively increasing workload exercise protocol (ramp) was set to achieve peak exercise in ~ 10 min [28]. CPET was interrupted when the subjects stated that they had reached maximal effort. Minute ventilation/carbon dioxide production (VE/VCO_2_) slope was calculated from the beginning of the loaded exercise to the respiration compensation point [29]. Percent of predicted for oxygen intake (VO_2_) and VE/VCO_2_ slope were calculated according to Hansen et al. [30] and Salvioni et al. [31].

### Statistical analysis

Statistical analysis was performed using SPSS 25.0 software (SPSS Inc, Chicago, IL, USA) and SAS version 9.4 (SAS Institute, Cary, NC, USA). Continuous variables were expressed as means ± standard deviation or median and [interquartile range] as appropriate, while discrete variables as absolute numbers and percentages. Comparisons between basal variables and end study variables were performed using paired t-tests or Wilcoxon signed rank test as appropriate. Chi-squared test was used to assess frequencies. Correlations were performed using Spearman’s rank correlation. The association between symptoms and major biological, anatomical and function parameters were analyzed by univariate logistic analysis. Symptoms significantly associated to assessed parameters were further evaluated adjusting for age, gender and previous cardiorespiratory diseases by a multivariate logistic model. Multicollinearity between variables was tested by VIF (variance inflation factor).

All tests were 2-sided. A p value ≤ 0.05 was considered as statistically significant.

The study was approved by the Ethics Committee of Centro Cardiologico Monzino and registered as R1174/20 CCM 1237.

## Results

**Fig. 1:**
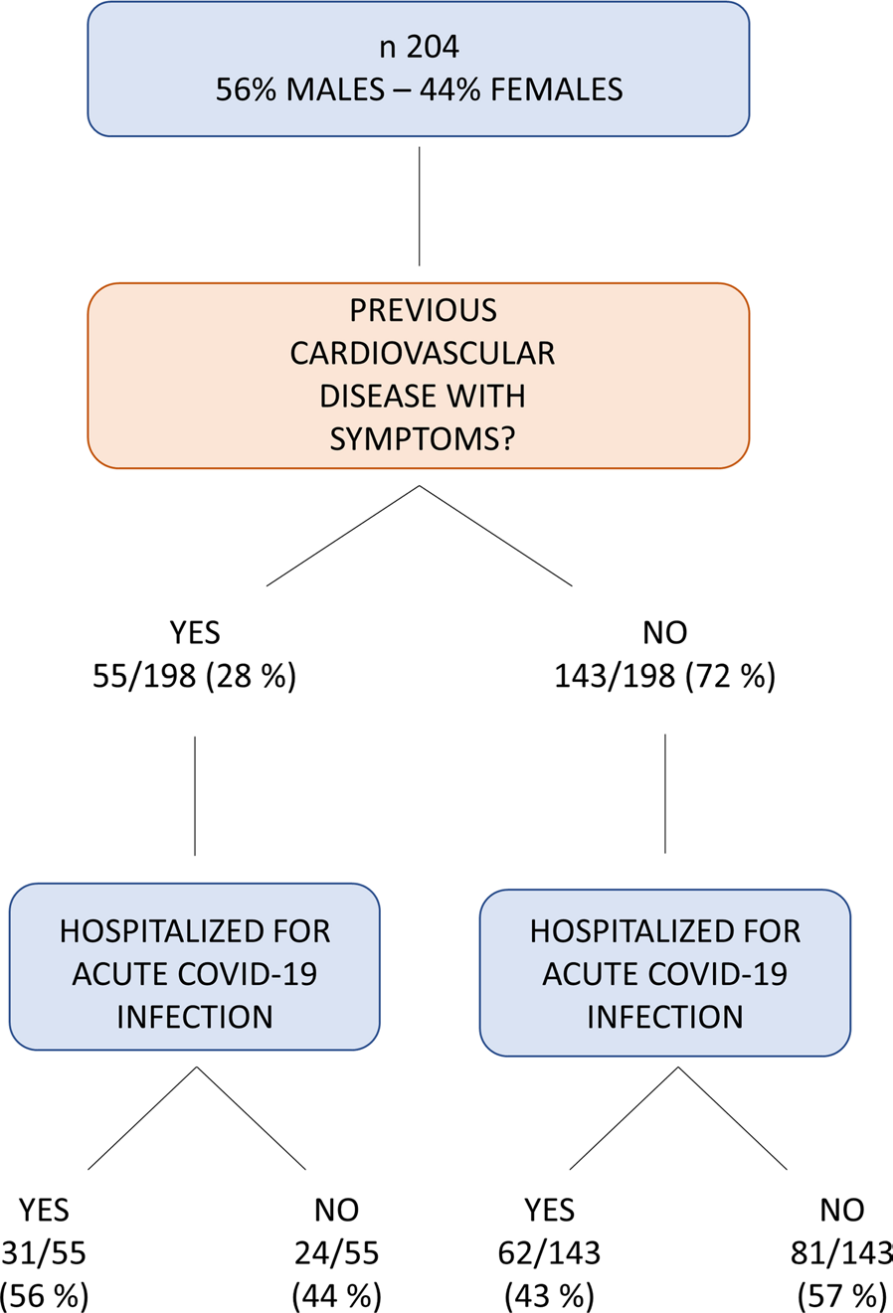
Composition of the population under analysis. The flow chart shows proportions of patients with previous cardiovascular disease and of patients hospitalized

We studied 204 post COVID-19 patients [age 56.5 ± 14.5 years, 89 females (44%), BMI 25.7 ± 4.0, 11 active smokers (6%)] referring a variety of cardio-respiratory symptoms. Main laboratory data are reported in Additional file 4: Table S1. One-hundred-fourteen patients had SARS CoV-2 acute infection including pneumonia between January and May 2020, 3 between June and August 2020, 87 between September 2020 and January 2021 and one in March 2021. Patients were evaluated 171 ± 85 days after the end of the acute COVID-19 infection which always included pneumonia. Diagnosis of pneumonia was based on clinical signs and symptoms, chest imaging (chest X-ray or CT scan) and laboratory data both in hospitalized and non-hospitalized patients. Fifty-five out of 198 cases had a previous not-severe cardiorespiratory disease with mild symptoms, while 143 referred good clinical conditions before the COVID-19 infection. In 6 cases the presence/absence of a well-defined pre-COVID-19 cardiorespiratory disease was not clear (Fig. 1). Ninety-three patients out of 204 recruited were hospitalized for acute COVID-19 syndrome either in general intensive care or dedicated acute SARS CoV-2 units. The presence/absence of previous cardiovascular disease did not influence the acute SARS CoV-2 infection hospitalization rate (p = 0.1). Major patients’ comorbidities and treatment are reported in Additional file 4: Table S2.

### Post COVID-19 symptoms

**Fig. 2:**
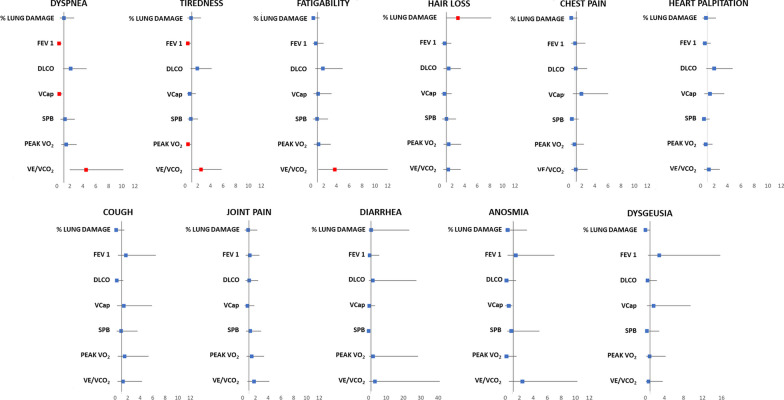
Relation between symptoms and the main cardiorespiratory variables. FEV1: forced expiratory volume in 1 s; DLCO: diffusing capacity of the lungs for carbon monoxide (data corrected for hemoglobin; Vcap: capillary volume; peak VO_2_: peak oxygen intake; VE/VCO_2_: minute ventilation/carbon dioxide production relationship slope

In 116 cases we have systematically administered and obtained a full answer to questionnaires. The great majority of patients referred more than 1 symptom and specifically 16% of patients referred 1 symptom, 20% 2 symptoms, 21% 3 symptoms and 43% > 3 symptoms. Dyspnea was reported in 45% of cases, cough in 11%, tiredness in 60%, chest pain in 19%, joint pain in 27%, heart palpitation in 26%, diarrhea 3%, anosmia in 6%, dysgeusia in 6%, exercise-induced fatigability in 77% and hair loss in 26%. Figure 2 shows the association between symptoms and major, arbitrarily chosen, biological, anatomical and function parameters: lung damage by CT scan, FEV_1_, DLCO, Vcap, SPB, peak VO_2_ and VE/VCO_2_ slope. Symptoms significantly associated with the above reported variables (dyspnea, exercise-induced fatigability, tiredness and hair loss) were further evaluated adjusting for age, gender and previous cardiorespiratory diseases. All associations remained statistically significant. Of note dyspnea, tiredness and fatigability were always associated to a high VE/VCO_2_ slope. Dyspnea was also associated with a low FEV_1_ and Vcap and tiredness with low FEV_1_ and peakVO_2_. Referred symptoms were further evaluated by grouping cases according to different variables (Additional figures) as need of SARS CoV-2 hospitalization, previous diagnosis of cardiorespiratory disease, lung damage at CT, CPET parameters, spirometry data and parameters analyzing the alveolar capillary membrane function. As cut-off values we used for lung damage at CT scan and SPB the median value and for all spirometry and CPET parameters < 80% of predicted value but VE/VCO_2_ slope where we considered as abnormal > 120% of predicted value. An abnormal value was observed in 10% of patients for FEV_1_, 12% for FVC, 39% for DLCO, 13% for DM, 36% for Vcap, 17% for VA, 50% for peakVO_2_ and 35% for VE/VCO_2_ slope. No differences except hair loss were observed between patients who had a more severe COVID-19 acute infection, as suggested by need of hospitalization, and those who did not need hospitalization (Additional file 1: Fig. S1a). Symptoms according to % of lung damage by CT scan (> 7.2% and ≤ 7.2%), to exercise performance (peak VO_2_ ≥ 80% vs. < 80% pred), and to ventilation efficiency during exercise (VE/VCO_2_ slope > 120% pred vs. ≤ 120% pred) are reported in Additional file 1: Fig. S1b, c and d, respectively. In patients with persistent higher lung involvement at CT scan, only hair loss was significantly more frequently observed. Patients with lower peakVO_2_ had more frequently tiredness, while those with higher VE/VCO_2_ slope reported dyspnea, tiredness and fatigability. Patients with FEV_1_, FVC and VA < 80% of predicted value were relatively few and no specific symptoms characterized these cases (Additional file 2: Fig. S2a, b, and c, respectively). No association was found between referred symptoms and previous cardiopulmonary disease (Additional file 2: Fig. S2d). The association between alveolar-capillary membrane function, and specifically DLCO, DM, Vcap and SPB, are reported in Additional file 3: Fig. S3a, b, c, and d, respectively. Grouping patients above and below 80% of predicted DLCO, DM and below and above the median value of SPB, no differences were referred to reported symptoms. Vice-versa subjects with lower Vcap had more frequently dyspnea and anosmia.

### Standard spirometry, alveolar capillary membrane function and SPB

Standard spirometry, lung diffusion and SPB data for the entire population and grouping patients according to previous cardiorespiratory disease, to lung damage at thoracic CT scan, to peak VO_2_ and VE/VCO_2_ are reported in Table 1. On the average DLCO was 22.2 ± 6.8 mL/min/mmHg (82 ± 19%pred), DM 47.6 ± 14.8 mL/min/mmHg (107 ± 23%pred) and Vcap 59 ± 17 mL (85 ± 22%pred). VA was 5.2 L [IQR 4.5–5.9], 93% pred [84–102]. In patients with DLCO < 80% (75 patients, 39%), VA, DM and Vcap were 84% [IQR 76–93], 89 ± 22% and 65 ± 17%, respectively. In patients with DLCO ≥ 80%, VA, DM and Vcap were 98% [IQR 91–106], 117 ± 17% and 96 ± 16%, respectively, (p < 0.001 for all). SPB (immature form) was 69.2 [IQR 46.9–98.2] AU in the entire population and 71.2 [IQR 45.8–114.2] AU and 66.1 [IQR 48.6–90.7] AU (p = 0.155) in patients with and without DLCO impairment, respectively. In our laboratory the SPB normal range for healthy subjects is 12 ± 6.5 AU. FEV_1_, FVC (both as %pred) and SPB but not lung diffusion parameters were more compromised in patients with previous cardiovascular diseases. Differently, both standard spirometry and lung diffusion parameters were more compromised in patients with greater lung parenchyma abnormalities at CT scan. Standard spirometry, DLCO (% pred), membrane diffusion (% pred) and capillary volume (absolute value), were associated to low peakVO_2_. Of note, a higher ventilatory inefficiency (high VE/VCO_2_ slope) was associated with borderline differences in standard spirometry and more relevant lung diffusion abnormalities including low Vcap.

**Table 1:** Spirometry (n = 200), DLCO (n = 194) and SPB (n = 194) values in patients with post COVID syndrome

	CP disease + (n 55)	CP disease − (n 143)	CT > 7.2% (n 51)	CT < = 7.2% (n 99)	peak VO2 < 80% (n 97)	peak VO2 > 80% (n 96)	VE/VCO2 > 120% (n 67)	VE/VCO2 < 120% (n 126)
Age (years)	53.8 ± 13.1	63.1 ± 16.1*	61.6 ± 13.2	54.1 ± 14.9*	53.2 ± 13.1	58.0 ± 14.8^#^	56.1 ± 16.0	54.9 ± 12.8
Gender (males)	37 (67%)	73 (51%)	29 (57%)	52 (53%)	62 (64%)	37 (36%)	37 (55%)	73 (58%)
BMI (kg/m^2^)	25.5 ± 3.8	25.9 ± 4.2	27.3 ± 3.9	25.1 ± 3.8*	26.1 ± 4.3	25.5 ± 3.6	26.3 ± 4.7	25.6 ± 3.6
SPB (AU)	84.0 [50.0–124.3]	66.1 [44.0–90.7]*	71.1 [52.5–114.2]	66.6 [47.7–88.4]	72.3 [48.5–110.6]	64.7 [43.6–84.6]^#^	69.2 [49.9–108.0]	67.0 [44.9–92.4]
FVC (%)	94 ± 19	101 ± 16*	93 ± 17	103 ± 17*	96 ± 17	104 ± 15*	97 ± 18	102 ± 16^#^
FEV1(%)	93 ± 20	101 ± 16*	95 ± 17	101 ± 16^#^	97 ± 17	104 ± 16*	97 ± 18	102 ± 15
FEV1/FVC	86.7 ± 15.9	86.2 ± 13.0	83.6 ± 14.5	88.6 ± 14.4^§^	85.2 ± 14.2	88.7 ± 13.1	85.2 ± 14. 8	87.7 ± 13.1
DLCO (mL/min/mmHg)	21.2 ± 7.2	22.3 ± 6.6	19. 8 ± 5.9	23.0 ± 6.7*	21.9 ± 7.2	22.9 ± 6.0	20.8 ± 6.7	23.3 ± 6.5^#^
DLCO (%)	80 ± 20	82 ± 19	77 ± 16	85 ± 20*	79 ± 21	86 ± 15^#^	77 ± 18	85 ± 18*
DLNO (mL/min/mmHg)	91.3 ± 32.9	95.2 ± 28.2	82.1 ± 26.5	99.7 ± 28.6*	93.3 ± 29.5	98.4 ± 28.0	91.2 ± 27.9	99.3 ± 28.1
DM (mL/min/mmHg)	45.5 ± 17.1	47.8 ± 13.7	41.5 ± 13.4	50.4 ± 14.3*	46.4 ± 15.4	49.8 ± 13.2	45.6 ± 15.0	49.5 ± 14.0
DM (%)	101 ± 23	107 ± 22	95 ± 20	113 ± 23*	102 ± 24	112 ± 20*	102 ± 22	110 ± 22^#^
Vcap (mL)	57 ± 18	60 ± 17	54 ± 17	62 ± 15*	58 ± 18	62 ± 15^#^	56 ± 18	62 ± 15^#^
Vcap (%)	82 ± 23	86 ± 22	81 ± 21	89 ± 22^§^	82 ± 23	88 ± 19	80 ± 22	88 ± 20^#^
VA (L)	5.2 [4.4–5.8]	5.2 [4.5–5.9]	5.0 [4.1–5.6]	5.3 [4.4–6.1]^@^	5.2 [4.5–6.2]	5.3 [4.5–5.8]	5.0 [4.3–5.8]	5.4 [4.6–6.2]

SPB: surfactant binding protein; AU: arbitrary unit; FVC: forced vital capacity; FEV1: forced expiratory volume in 1 s; DLCO: diffusing capacity of the lungs for carbon monoxide (data corrected for hemoglobin); DLNO: diffusing capacity of the lungs for nitric oxide; DM: membrane diffusion; Vcap: capillary volume; VA: alveolar volume; CP disease: history of cardiopulmonary diseases; CT: thoracic computer tomography; peak VO_2_: peak oxygen intake; VE/VCO_2_: minute ventilation/carbon dioxide production relationship. *p < 0.01; ^#^p < 0.05; ^§^p = 0.05; ^@^p = 0.06

### CT scan

CT scan (150 cases) showed a residual lung parenchyma damage of 7.2 ± 3.2% of lung tissue. Of note the amount of residual lung parenchyma damage was 6.7 ± 2.1% and 8.6 ± 4.7% (p = 0.018) and 6.8 ± 2.5% and 7.6 ± 3.7% (p = 0.092) in patients with previous cardiovascular disease with symptoms and SARS CoV-2 hospitalization, respectively.

### Transthoracic echocardiography

Cardiac ultrasound showed preserved right and left ventricle geometry, dimension and function (Additional file 4: Table S3). Right and left heart dimension, albeit within normal range, were greater in patients with previous cardiovascular diseases while TAPSE was lower. Similarly, in these patients mitral and tricuspid regurgitation albeit of minor severity were greater. However, no major differences were observed among the most of the TTE parameters grouping the patients according to CT parenchymal involvement, to reduced/preserved peak VO_2_, or to normal/reduced VE/VCO_2_ slope. Minor but significant lower left ventricle diastolic performance (E/A) value was observed in subjects with more compromised lung parenchyma, while TAPSE and tricuspid regurgitation were lower and higher, respectively, in subjects with reduced peak VO_2_.

### Cardiopulmonary exercise test

CPETs were maximal or near maximal in the vast majority of cases (RQ = 1.08 [1.04–1.13]). On the average the ramp protocol was 13.5 ± 4.6 Watts/min. Cardiopulmonary exercise data showed a slightly impaired exercise performance (peakVO_2_ = 84 ± 18%pred) and a normal ventilation efficiency as shown by the VE/VCO_2_ slope = 29.7 [26.0–32.4] or 112 [102–123] % pred as normal was the VE intercept 3.38 ± 2.68 on the VE/VCO_2_ relationship. A respiratory limitation defined as a breathing reserve < 20% was observed only in 5 cases, two patients had a O_2_ saturation at peak < 90% while an erratic breathing pattern was observed in 3 cases. CPET data grouping patients according to previous cardiorespiratory disease, to lung damage at thoracic CT scan, and to DLCO and Vcap are reported in Table 2. Previous cardiorespiratory disease was associated with lower VO_2_ at peak and anaerobic threshold and higher VE/VCO_2_. Presence of lung damage at CT scan revealed differences in peak heart rate and in VO_2_ at peak exercise and anaerobic threshold but only as absolute value (mL/min/kg) and not as a percentage of predicted value. Low DLCO was associated with a worse exercise performance both in terms of peakVO_2_ and VE/VCO_2_. Of note, VE/VCO_2_ Y-intercept value was unaffected by DLCO as well as by presence/absence of previous cardiorespiratory disease or entity of lung damage. Abnormal DLCO and both its components were associated to a reduced peak exercise HbO_2_ saturation. DM was ≤ 80% of predicted value in 24 cases and > 80% in 157. In patients with DM ≤ 80% of predicted VO_2_ at peak, AT was lower compared to patients with DM > 80% of predicted as lower was peak SpO_2_ and heart rate.

**Table 2:** Cardiopulmonary exercise test data in 193 patients with post COVID syndrome

	CP disease + (n 55)	CP disease − (n 143)	CT > 7.2% (n 51)	CT < = 7.2% (n 99)	DLCO < 80% (n 75)	DLCO > 80% (n 119)	Vcap < 80% (n 63)	Vcap > = 80% (n 114)
Age (years)	53.8 ± 13.1	63.1 ± 16.1*	61.6 ± 13.2	54.1 ± 14.9*	58.8 ± 15.6	54.1 ± 13.2^#^	59.0 ± 14.8	54.1 ± 13.6^#^
Gender (males)	37 (67%)	73 (51%)	29 (57%)	52 (53%)	39 (52%)	70 (59%)	34 (54%)	66 (58)
BMI (kg/m^2^)	25.5 ± 3.8	25.9 ± 4.2	27.3 ± 3.9	25.1 ± 3.8*	26.5 ± 4.6	25.2 ± 3.6^#^	26.4 ± 4.6	25.3 ± 3.8
Ramp protocol (Watt/min)	12.6 ± 4.5	13.9 ± 4.7	12.7 ± 4.5	13.7 ± 4.5	12.4 ± 4. 38	14.3 ± 4.8*	13.4 ± 4.1	13.9 ± 4.9
Peak HR (bpm)	127 ± 27	145 ± 26*	131 ± 28	143 ± 27^#^	133 ± 29	145 ± 25*	131 ± 28	146 ± 25*
Respiratory exchange ratio	1.08 [1.03–1.16]	1.08 [1.04–1.13]	1.07 [1.03–1.14]	1.08 [1.04–1.12]	1.08 [1.02–1.14]	1.09 [1.05–1.13]	1.07 [1.02–1.13]	1.09 [1.05–1.14]
Peak power (Watt)	104 [84–158]	132[97–165^§^]	112 [85–156]	124 [88–169]	112 [82–147]	136 [99–177]*	130 [97–165]	130 [96–165]
Peak VO_2_ (mL/min/kg)	19.3 ± 5.9	22.3 ± 6.3*	19.6 ± 5.4	22.0 ± 6.1^#^	19.1 ± 5.5	23.4 ± 6.4*	20.0 ± 5.4	23.0 ± 6.7*
Peak VO_2_ (% pred)	73.0 ± 17.33	83.5 ± 17.9*	80.6 ± 17.6	80.7 ± 18.6	76.3 ± 18.3	84.1 ± 18.3	78.1 ± 18.7	83.4 ± 18.0
VO_2_/work (mL/min/Watt)	9.6 ± 1.2	9.9 ± 1.3	9.8 ± 1.4	9.8 ± 1.3	9.7 ± 1.3	9.9 ± 1.3	9.6 ± 1.1	9.9 ± 1.4
VE intercept (L/min)	3.15 ± 2.55	3.38 ± 2.59	3.63 ± 2.82	3.06 ± 2.41	3.31 ± 2.97	3.42 ± 2.55	3.91 ± 3.02	3.25 ± 2.53
VE/VCO_2_ slope	31.8 [28.6–35.2]	29.3 [25.8–31.6]*	29.7 [27.3–32.5]	29.6 [25.9–32.3]	30.8 [26.3–34.6]	29.1 [25.8–31.3]*	30.1 [25.4–34.6]	29.4 [26.0–31.6]
VO_2_/HR (mL/beat)	11.3 [9.4–13.7]	11.0 [8.6–13.8]*	11.8 [9.3–13.7]	10.4 [8.5–13.7]	10.5 [8.6–12.1]	11.7 [9.3–14.0]^#^	11.4 [9.5–13.8]	11.3 [8.7–13.8]
VO_2_/HR (% pred)	97.6 ± 19.5	99.4 ± 18.3	99 [87.5–112]	99.5 [86–110]	94 [83–106]	101 [89–115]	97.5 [86–111]	100 [87–112]
AT VO2 (mL/min/kg)	11.6 [9.9–15.4]	13.4 [11.6–16.6]^#^	12.2 [11.2–14.1]	13.8 [11.5–16.7]^#^	11.9 [9.6–14.6]	14.0 [11.8–17.1]*	12.8 [10.4–15.3]	13.8 [11.4–17.2]^#^
Rest SpO_2_ (%)	97.0 ± 1.5	97.4 ± 1.3	97.1 ± 1.3	97.4 ± 1.4	97.2 ± 1.3	97.3 ± 1.4	97.12 ± 1.4	97.4 ± 1.3
Peak SpO_2_ (%)	96.8 ± 2.1	97.1 ± 1.6	96.8 ± 2.0	97.1 ± 1.4	96.5 ± 2.1	97.4 ± 1.2*	96.6 ± 2.0	97.3 ± 1.2*
Peak VE (L/min)	58.4 [46.7–70.9]	57.6 [46.4–70.9]	57.4 [50.0–69.0]	57.4 [43.9–70.4]	55.2 [43.9–66.5]	62.9 [49.0 -76.1]^#^	57.3 [50.2–75.8]	59.3 [46.1–72.0]
Peak RR (breath/min)	33.7 ± 7.3	32.2 ± 7.0	33.5 ± 7.2	31.9 ± 6.7	32.4 ± 6.7	32.7 ± 7.3	31.7 ± 7.1	32.9 ± 6.9
VT (L)	1.7 [1.4–2.4]	1.8 [1.5–2.4]	1.8 [1.5–2.2]	1.8 [1.4–2.3]	1.7 [1.4–2.1]	1.9 [1.5–2.5]^#^	1.9 [1.6–2.4]	1.8 [1.5–2.4]
Breathing Reserve (%)	48.76 ± 14.24	50.63 ± 11.79	46.91 ± 15.88	51.52 ± 10.99^#^	50.25 ± 13.38	49.97 ± 11.93	50.88 ± 13.2	50.34 ± 11.33
peak SBP (mmHg)	183 ± 30	193 ± 28§	192 ± 28	188 ± 31	187 ± 28	192 ± 30	187 ± 28	193 ± 30

HR: heart rate; peak VO_2_: peak oxygen intake; VE: ventilation; VE/VCO_2_ slope: minute ventilation/carbon dioxide production relationship; SpO_2_: peripheral oxygen saturation; RR: respiratory rate; VT: tidal volume; SBP: systolic blood pressure; CP disease: history of cardiopulmonary diseases; CT: thoracic computer tomography; DLCO: diffusing capacity of the lungs for carbon monoxide (data corrected for hemoglobin); Vcap: capillary volume; DM: membrane diffusion. *p < 0.01; ^#^p < 0.05; ^§^p = 0.05; ^@^p = 0.06

### Correlations

Several correlations were tested; on the average correlations were albeit significant weak (Table 3).

**Table 3:** Correlation between major cardiorespiratory parameters in 204 patients with post COVID syndrome

	SPB (AU)	DLCOcHb (mL/min/mmHg)	DM (mL/min/mmHg)	Vcap (mL)	VA (L)	peak VO2 (mL/min/kg)	VE/VCO2 slope
% of lung damage	0.161 (p = 0.053)	− 0.248**	− 0.222**	− 0.284**	− 0.176*	− 0.209*	0.064
SPB (AU)		− 0.244**	− 0.169*	− 0.325**	− 0.079	− 0.214**	0.145*
DLCOcHb (mL/min/mmHg)			0.914**	0.848**	0.799**	0.573**	− 0.364**
DM (mL/min/mmHg)				0.675**	0.769**	0.603**	− 0.331**
Vcap (mL)					0.558**	0.465**	− 0.260**
VA (L)						0.459**	− 0.331**
peak VO_2_ (mL/min/kg)							− 0.479**

SPB: surfactant binding protein; AU: arbitrary unit; DLCOcHb: diffusing capacity of the lungs for carbon monoxide (data corrected for hemoglobin); DM: membrane diffusion; Vcap: capillary volume; VA: alveolar volume; peak VO_2_: peak oxygen intake; VE/VCO_2_ slope: minute ventilation/carbon dioxide production relationship

*p < 0.05; **p < 0.01

## Discussion

In the present study we applied a holistic approach to assess cardiorespiratory function in patients with post COVID-19 syndrome. We showed that dyspnea, tiredness and fatigability, but not other typical post COVID symptoms, are associated to inefficiency of ventilation during exercise. As regards alveolar capillary membrane, abnormalities are mainly due to Vcap reduction supporting the hypothesis of primary role of endothelial/vascular dysfunction in post COVID syndrome. Moreover, SPB was fivefold higher than in normal subjects showing that the alveolar capillary membrane is under a long term remodeling process which includes abnormal cell function.

The population we studied is characterized by a variety of symptoms with tiredness, exercise-induced fatigability and dyspnea, present in ~ 60%, 75% and 45% of cases. A few authors [1, 32–35] previously reported in similar populations that standard spirometry was within normal ranges, mean lung diffusion was on the lower end of normality and CPET data characterized by minor peakVO_2_ reduction and a minimal increase in ventilation inefficiency. Our data confirm these findings, which can be attributed to cardiorespiratory mild impairment but also to deconditioning associated with prolonged inactivity. Of note the presence of pre COVID cardiorespiratory disease with mild symptoms did not influence post COVID symptoms albeit affecting standard spirometry and CPET data. Moreover, referred symptoms were not related to lung parenchyma involvement at CT, to severity of COVID-19 infection or to peakVO_2_ but were related to VE/VCO_2_ slope, a parameter directly associated to the way patients breathes.

In the present study we deepened our analysis on alveolar capillary membrane function showing a significant involvement of the alveolar capillary membrane. Specifically, DLCO impairment was mainly associated to Vcap impairment [36]. Indeed, the relevant reduction of Vcap was partially compensated by a preserved DM so that overall DLCO was only minimally impaired. It must be underlined, however, that the calculation of absolute and the percent of predicted values for DM and Vcap underwent a few changes in the last years. We applied those derived from the original ERS report for DLCO and DM and for the update ERS statement for Vcap [22]. We did so because the DLCO and DM we report are the most frequently used in the literature while the Vcap, which is more rarely analyzed, is definitively more correct if calculated applying the updated ERS document. Accordingly, we might have overestimated the DM. Our observation is in line with a few reports which suggest a major clinical role in thrombosis and vasculopathy in the genesis of the post COVID-19 syndrome [10, 37, 38]. In the present study impaired Vcap was significantly associated with dyspnea even after adjustments for confounding variables while total lung diffusion and membrane diffusion were not. In parallel, SPB blood value was significantly increased, albeit not associated to specific symptoms. Finally, it must be underlined that a strong correlation was observed between exercise performance parameters, both peakVO_2_ and VE/VCO_2_ slope, and DLCO and its two components, DM and Vcap suggesting a link between abnormalities in exercise performance and alveolar-capillary membrane function in post COVID patients. The Y-axis intercept values on the VE/VCO_2_ relationship were in the normal range and not influenced by the presence of previous cardiorespiratory disease, by COVID-19 infection severity, by the amount of lung damage or alveolar capillary membrane function. This finding confirms the absence of relevant dead space at rest as observed in patients with obstructive lung diseases [39, 40].

SPB data deserve a detailed discussion. Indeed, SPB immature form has been proposed as a biological marker of the alveolar capillary membrane impairment severity and dysregulation [41, 42]. SPB is produced only by alveolar cells and, in normal conditions, it is not found in the blood stream. Its presence indicates a damage of the alveolar capillary membrane, so that the greatest is the amount of blood SPB immature form the greatest is the membrane damage [43]. SPB has also a strong prognostic capability in heart failure and it changes along with heart failure severity [43, 44]. The present is the first report on SPB values in the post COVID-19 syndrome. We observed median values more than 5 times greater than in normal subjects and related to severity of COVID-19 infection and lung diffusion impairment. Again, as regards lung diffusion the highest correlation was found between Vcap and SPB confirming the hypothesis that COVID 19 pneumonia severely damages the pulmonary vasculature and, according to the present data, also influences the post COVID syndrome as part of the long term alveolar-capillary membrane healing process.

The dimensions and performance of the four heart chambers were all preserved confirming the great capability of the right heart to support an increased hemodynamic burden. Similarly exercise performance was, on the average, preserved in patients with post COVID-19 syndrome albeit influenced by previous cardiovascular diseases. As regard the observed correlation between DLCO and exercise performance it should be underlined that in the present setting both DM and Vcap abnormalities play a pivotal role [41, 42].

All-together the present data suggest that in the post COVID-19 syndrome a central role is played by inefficiency of ventilation and, at the alveolar capillary membrane level, by reduction of Vcap. The high SPB value shows a dysregulation of alveolar cells. It is at present totally unknown whether with time Vcap can be restored in post COVID-19 syndrome albeit the presence of an elevated immature SPB value suggests a dynamic status of the alveolar capillary membrane with dysfunction of the alveolar cells. Studies are needed to assess the long term behavior of post COVID symptoms and function abnormalities including lung parenchyma damage, standard spirometry, alveolar capillary function, SPB and cardiopulmonary exercise test data.

The present study has relevant limitations. First of all, the population we studied was referred to a cardiopulmonary laboratory and therefore it is a selected population. Secondly, our report is based on the description of several tests and the correlations described do not imply any cause-effect relationship. Thirdly, patients underwent to a relevant number of tests so that the study was very demanding for patients. Accordingly, only 86 patients performed all the tests. Indeed, CT scan was evaluated in 150 patients since a few subjects presented a CT scan not assessable by our automatic reading system. Similarly, we evaluated only completed questionnaires which were available in 57% of cases. Two reasons are behind this: (a) the a priori protocol choice to leave subjects alone when writing the questionnaires avoiding any possible external interference and (b) a complete symptoms questionnaire was utilized only after November 2020 (n = 116), when the existence of the long term COVID was recognized. Furthermore, it must be acknowledged that the administered questionaries’ evaluated only presence/absence of symptoms but not their intensity. It must be underlined that some symptoms such cough, anosmia, dysgeusia and diarrhea were rarely observed in the present population limiting the possibility to identify variables associated with these symptoms. Moreover, we assessed the severity of acute COVID 19 grouping patients according to the need of hospitalization. It is recognized that this criterion is questionable and subjected to variability. However, particularly in 2020 COVID 19 pandemia was extremely severe in Lombardia and we have no better way to assess COVID 19 severity [45]. Finally, different formulas for the best prediction of DLCO/DLNO, DM and Vcap are still under investigation in the scientific community with a never-ending debate. In any case we used formulas which are associated, if anything, with an overestimation of DM and Vcap values reinforcing our findings.

In conclusion in patients with post COVID-19 syndrome cardiorespiratory symptoms are linked to respiration modalities during exercise. In these patients the alveolar cells are dysregulated with a relevant reduction of Vcap but not of DM so that overall diffusion is only minimally impaired.

### Supplementary Information

**Additional file 1: Figure S1.** Referred symptoms according to: a. need of SARS CoV-2 hospitalization, b. lung damage at CT, cardiopulmonary test parameters (c. peakVO_2_ and d. VE/VCO_2_ slope). CT: thoracic computer tomography; peak VO_2_: peak oxygen intake; VE/VCO_2_: minute ventilation/carbon dioxide production relationship.

**Additional file 2: Figure S2.** Referred symptoms according to spirometry data (a. FEV1, b. FVC and c. VA) and previous diagnosis of cardiorespiratory disease (d). FEV1: forced expiratory volume in 1s; FVC: forced vital capacity; VA: alveolar volume.

**Additional file 3: Figure S3.** Referred symptoms according to alveolar capillary membrane function parameters: a. DLCO, b. DM, c. Vcap and d. SPB. DLCO: diffusing capacity of the lungs for carbon monoxide; DM: membrane diffusion; Vcap: capillary volume; SPB: surfactant binding protein.

**Additional file 4: Table S1.** Main laboratory data. **Table S2.** Major comorbidities and chronic cardiovascular therapy. **Table S3.** Major cardiac ultrasound data (n = 196).

## Data Availability

Raw data will be available upon request at www.zenodo.org.

## Abbreviations

CPET: Cardiopulmonary exercise test
CT: Thoracic computer tomography
DLCO: Carbon monoxide
DLNO: Nitric oxide lung diffusion
DM: Membrane diffusion
FEV_1_: Forced expiratory volume
HAAs: High attenuation areas
SPB: Surfactant derived protein B
TTE: Echocardiography
VA: Alveolar volume
Vcap: Capillary volume
VE/VCO_2_: Minute ventilation/carbon dioxide production
VO_2_: Oxygen intake
