# Supplementary figures and images for: Symptomatic post COVID patients have impaired alveolar capillary membrane function and high VE/VCO2
Source: Respir Res. 2024 Feb 8;25:82. doi: 10.1186/s12931-023-02602-3 (PMC10851544; doi:10.1186/s12931-023-02602-3)

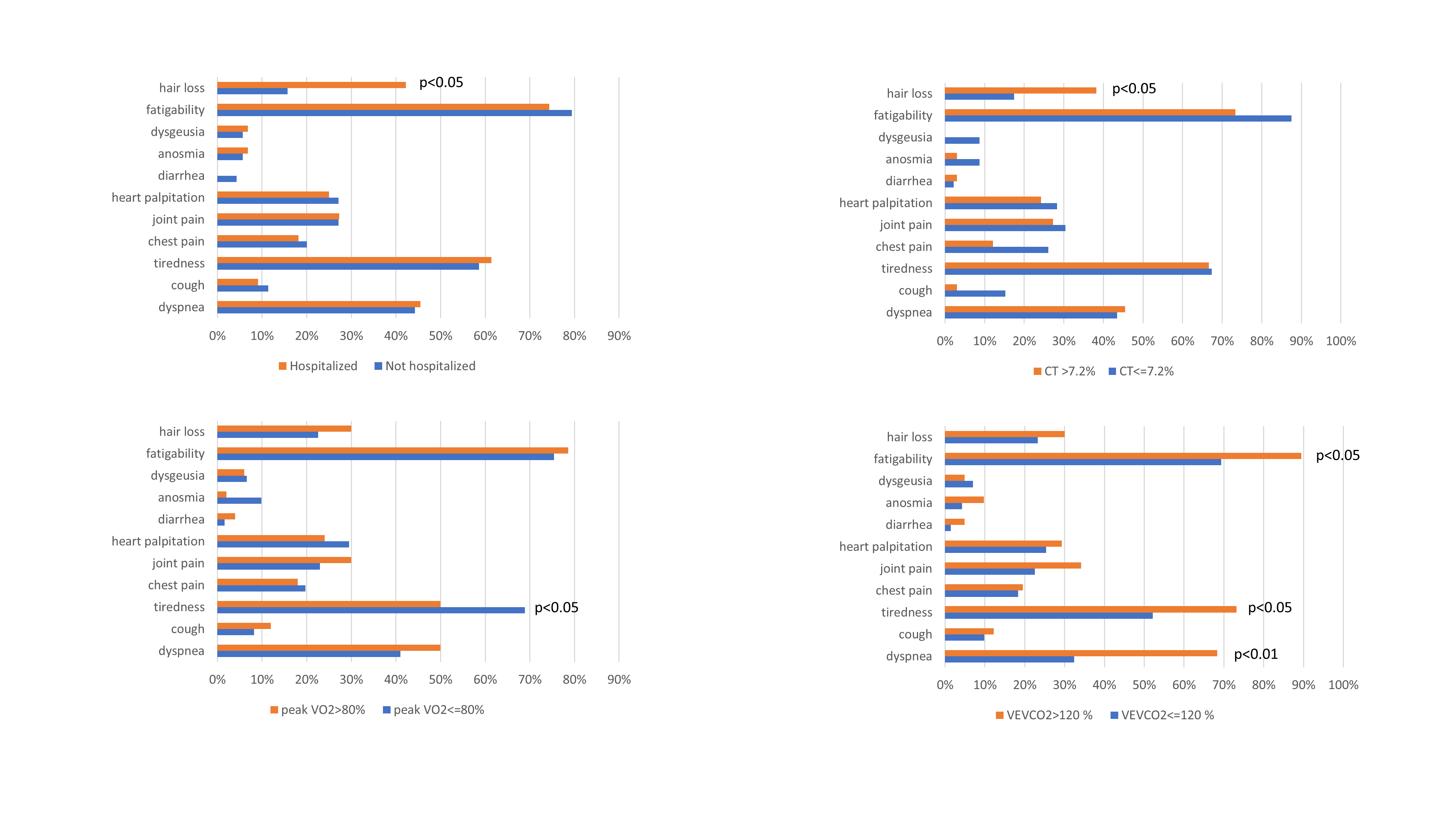

Supplement: Supplementary file 1 — Additional file 1: Figure S1. Referred symptoms according to: a. need of SARS CoV-2 hospitalization, b. lung damage at CT, cardiopulmonary test parameters (c. peakVO2 and d. VE/VCO2 slope). CT: thoracic computer tomography; peak VO2: peak oxygen intake; VE/VCO2: minute ventilation/carbon dioxide production relationship. [file 12931_2023_2602_MOESM1_ESM.tif]

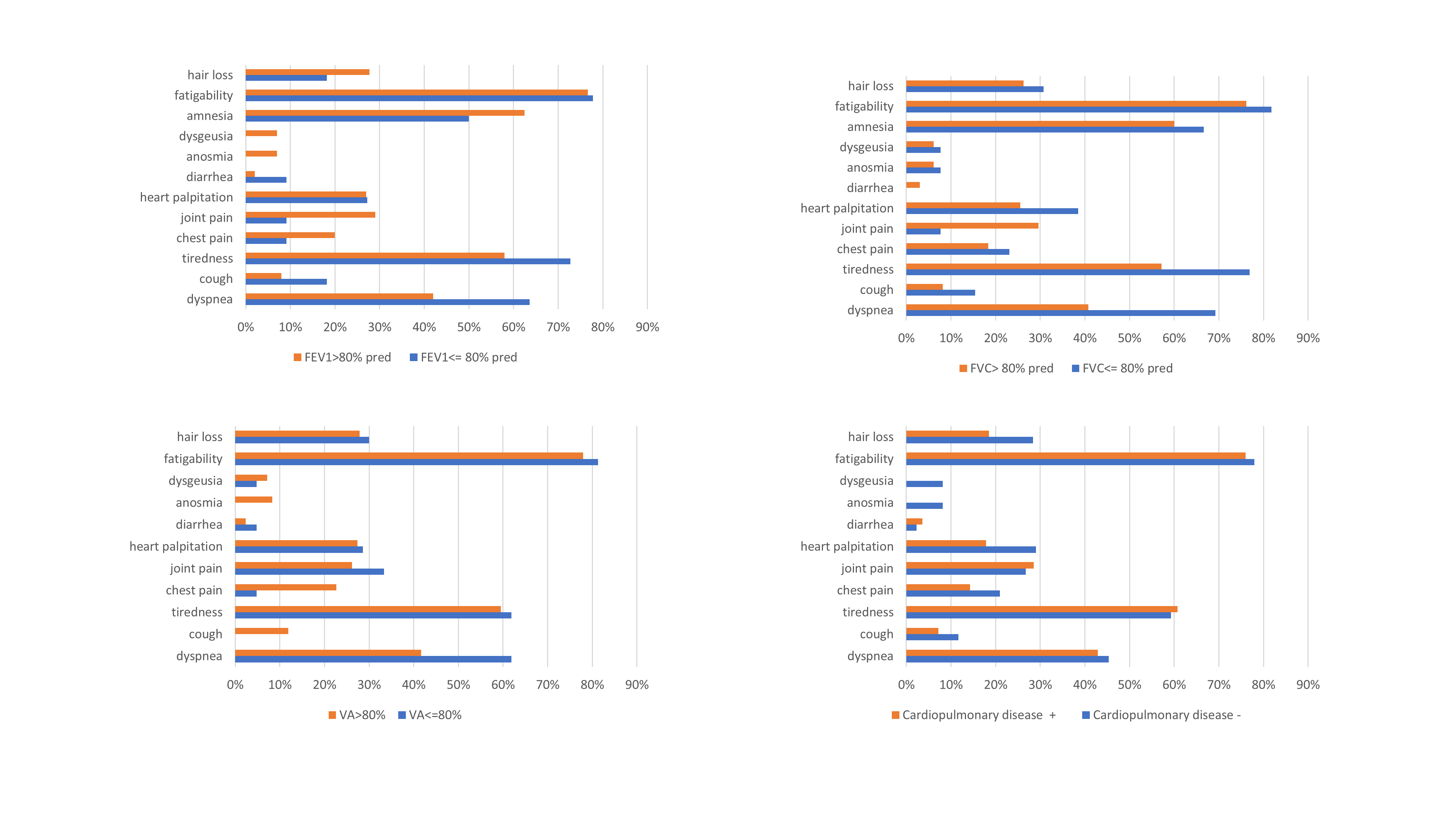

Supplement: Supplementary file 2 — Additional file 2: Figure S2. Referred symptoms according to spirometry data (a. FEV1, b. FVC and c. VA) and previous diagnosis of cardiorespiratory disease (d). FEV1: forced expiratory volume in 1s; FVC: forced vital capacity; VA: alveolar volume. [file 12931_2023_2602_MOESM2_ESM.tif]

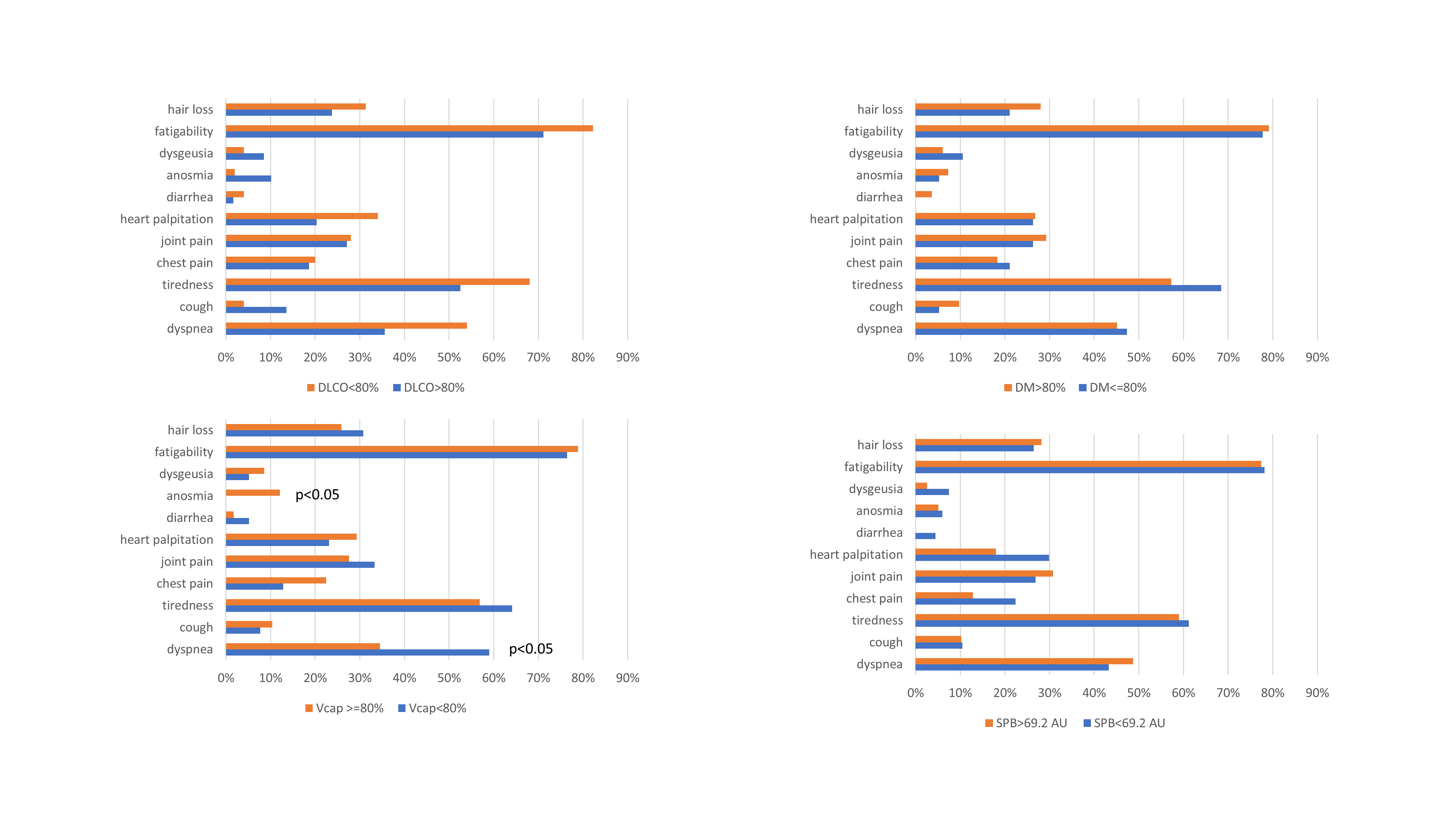

Supplement: Supplementary file 3 — Additional file 3: Figure S3. Referred symptoms according to alveolar capillary membrane function parameters: a. DLCO, b. DM, c. Vcap and d. SPB. DLCO: diffusing capacity of the lungs for carbon monoxide; DM: membrane diffusion; Vcap: capillary volume; SPB: surfactant binding protein. [file 12931_2023_2602_MOESM3_ESM.tif]
