# Supplementary material for: Symptomatic post COVID patients have impaired alveolar capillary membrane function and high VE/VCO2
Source: Respir Res. 2024 Feb 8;25:82. doi: 10.1186/s12931-023-02602-3 (PMC10851544; doi:10.1186/s12931-023-02602-3)
Supplement: Supplementary file 4 — Additional file 4: Table S1. Main laboratory data. Table S2. Major comorbidities and chronic cardiovascular therapy. Table S3. Major cardiac ultrasound data (n = 196). [file 12931_2023_2602_MOESM4_ESM.docx]

Table S1: Main laboratory data.

| Blood samples | |  | Normality ranges |
| --- | --- | --- | --- |
| Leucocytes (10^3/uL) | 6.7 [6-7.8] |  | [4.0 - 11.0] |
| Neutrophils (%) | 59 [52.8-64.1] |  | [37.0 - 73.0] |
| Limphocytes (%) | 30.15±9.04 |  | [20.0 - 45.0] |
| Monocytes (%) | 7.88±2.59 |  | [2.5 - 10.0] |
| Eosinophils (%) | 1.8 [1.1-2.8] |  | [< 5.0] |
| Platelets (10^3/uL) | 235 [200-280] |  | [130 - 400] |
| RDW (CV%) | 12.9 [12.4-13.6] |  | [11.5 - 14.5] |
| Hemoglobin (g/dL) | 14 [13.0-15.0] |  | M[13.5 - 17.5]/F[12.0 - 15.8] |
| MCV (fL) | 86.3±7.0 |  | [80.0 - 94.0] |
| Urea (mg/dL) | 37 [32-45] |  | [10 - 50] |
| Creatinine (mg/dL) | 0.93±0.23 |  | [0.61 - 1.24] |
| Na+ (mmol/L) | 140.09±2 |  | [135 - 146] |
| K+ (mmol/L) | 4.16±0.33 |  | [3.50 - 5.10] |
| ALT (UI/L) | 21 [16-28] |  | [6 - 59] |
| AST (UI/L) | 24±8 |  | [5 - 35] |
| High sensitiveTroponin I (ng/L) | 9.51±31.7 |  | [< 53.53] |
| D-dimer (ng/mL) | 414±396 |  | [< 600] |
| Ferritine (ng/mL) | 123±126 |  | [22 - 322] |
| CRP (mg/L) | 2.2±5.9 |  | [0.0 - 7.5] |
| PCT (ng/mL) | 0.02±0.03 |  | [< 0.5] |
| BNP (pg/mL) | 52±105 |  | [10 - 100] |
| ST-2 (ng/mL) | 16.62±10.02 |  | [< 37.0] |
| LDH (U/L) | 189±33 |  | [135 - 225] |
| Arterial blood samples | |  |  |
| pH | 7.43±0.03 |  | [7.35-7.45] |
| pCO2 (mmHg) | 39.2±8.29 |  | [35.0-48.0] |
| pO2 (mmHg) | 94 [87-101] |  | [83-108] |
| HCO3- (mmol/L) | 25±2 |  | [21-28] |
| Lactates (mmol/L) | 0.9 [0.6-1.3] |  | [-2-3] |
| Glucose (mg/dL) | 105 [96-114.5] |  | [79-110] |
| Saturation (%) | 98.6±1.2 |  | [94-98] |

RDW: red blood cell distribution width; MCV: mean corpuscular volume; Na+: sodium; K+: potassium; ALT: alanine transaminase; AST: aspartate transferase; CRP: C-reactive protein; PCT: procalcitonin; BNP: B-type natriuretic peptide; ST-2: interleukin ST-2; LDH: lactate dehydrogenase; pCO_2_: partial pressure of carbon dioxide; pO_2_: partial pressure of oxygen; HCO3-: bicarbonate

Table S2: Major comorbidities and chronic cardiovascular therapy.

|  | n (%) | |
| --- | --- | --- |
| Respiratory disease | 18 | (9%) |
| Arterial hypertension | 66 | (32%) |
| Atrial fibrillation | 16 | (8%) |
| Type II diabetes mellitus | 15 | (7%) |
| Dyslipidemia | 41 | (20%) |
| Previous myocardial infarction | 20 | (10%) |
| Heart failure | 11 | (5%) |
|  |  |  |
| Aspirin | 41 | (20%) |
| Second antiplatelet drug | 15 | (7%) |
| Anticoagulant therapy | 13 | (6%) |
| Beta-blocker | 49 | (24%) |
| Angiotensin-converting enzyme inhibitor | 19 | (9%) |
| Angiotensin receptor blocker | 21 | (10%) |
| Sacubitril/valsartan | 7 | (3%) |
| Mineralocorticoid receptor antagonist | 10 | (5%) |
| Diuretic | 23 | (11%) |
| Oral antidiabetic | 14 | (7%) |
| Statin | 42 | (21%) |

Table S3: Major cardiac ultrasound data (n = 196).

|  | **CP disease +**  **(n 55)** | **CP disease –**  **(n 143)** | **CT>7.2%**  **(n 51)** | **CT<=7.2%**  **(n 99)** | **peak VO_2_<80%**  **(n 97)** | **peak VO_2_>80%**  **(n 96)** | **VE/VCO_2_ slope>120%**  **(n 67)** | **VE/VCO_2_ slope<120%**  **(n 126)** |
| --- | --- | --- | --- | --- | --- | --- | --- | --- |
| **Age (years)** | 53.8±13.1 | 63.1±16.1* | 61.6±13.2 | 54.1±14.9* | 53.2±13.1 | 58.0±14.8^#^ | 56.1±16.0 | 54.9±12.8 |
| **Gender (males)** | 37 (67%) | 73 (51%) | 29 (57%) | 52 (53%) | 62 (64%) | 37 (36%) | 37 (55%) | 73 (58%) |
| **BMI (kg/m2)** | 25.5±3.8 | 25.9±4.2 | 27.3±3.9 | 25.1±3.8* | 26.1±4.3 | 25.5±3.6 | 26.3±4.7 | 25.6±3.6 |
| **EDV (mL)** | 99.0 [82.0-129.5] | 93.0 [76.5-114.5]# | 93.0 [79.0-114.5] | 89 [75-112] | 99.0 [82.0-119.3] | 95 [77-116] | 93.0 [78.0-116.5] | 96.0 [82.0-117.0] |
| **ESV (mL)** | 39.0 [30.0-59.5] | 34.0 [28.5-43.5]# | 36.0 [31.0-43.0] | 34.0 [26.5-45.5] | 38.0 [29.8-48.3] | 34 [29-44] | 38.0 [46.5-29.0] | 36 [29-46] |
| **LVEF (%)** | 61.0 [49.1-65.9] | 62.3 [58.1-66.1]# | 62.8 [56.4-66.1] | 61.8 [56.4-65.8] | 61.9 [54.7-65.6] | 62.3 [58.4-66.1] | 61.4 [53.8-65.5] | 62.3 [58.5-66.0] |
| **LAV ind (mL/m^2^)** | 33.1±15.9 | 25.3±7.8* | 27.9±10.9 | 25.1±8.8 | 27.5±10.6 | 26.7±8.3 | 27.0±11.2 | 27.0±8.5 |
| **MR** | 1.0 [0.5-1.0] | 0.5 [0.0-1.0]* | 0.5 [0.0-1.0] | 0.5 [0.0-1.0] | 0.5 [0.0-1.0] | 0.5 [0.0-1.0] | 0.5 [0.0-1.0] | 0.5 [0.0-1.0] |
| **E/A** | 0.98 [0.70-1.64] | 1.02 [0.81-1.20] | 0.92 [0.70-1.08] | 1.04 [0.83-1.38]* | 1.00 [0.77-1.25] | 1.03 [0.82-1.21] | 0.98 [0.78-1.18] | 1.07 [0.81-1.22] |
| **E/e'** | 8.0 [6.0-12.0] | 7.5 [6.0-9.0]# | 8 [7-10] | 8 [6-9] | 8 [6-10] | 7.4 [6.0-9.0] | 7.0 [6.0-9.5] | 8 [6-9] |
| **RVAd (cm^2^)** | 18.0±5.2 | 16.9±4.0 | 16.7±3.57 | 17.0±4.6 | 17.0±4.6 | 17.4±4.3 | 16.8±4.6 | 17.3±4.4 |
| **FAC (cm^2^)** | 48.5±8.6 | 48.6±9.1 | 48.6±9.9 | 48.4±8.9 | 48.2±8.2 | 48.2±9.4 | 48.8±8.2 | 47.8±9.1 |
| **TAPSE (mm)** | 22.9±5.2 | 24.3±3.6# | 24.6±4.5 | 23.3±3.6@ | 23.1±4.1 | 24.6±3.9 | 23.6±4.0 | 24.0±4.1 |
| **RA area (cm^2^)** | 16.9±4.9 | 15.2±9.4 | 17.5±15.2 | 14.5±4.0 | 15.0±3.9 | 14.9±3.7 | 14.8±4.0 | 15.0±3.6 |
| **TR** | 1.0 [0.5-1.0] | 0.5 [0.5-1.0]* | 1.0 [0.5-1.0] | 1.0 [0.5-1.0] | 1.0 [0.5-1.0] | 0.5 [0.5-1.0]* | 1.0 [0.5-1.0] | 1.0 [0.5-1.0] |
| **PPS (mmHg)** | 29.9±9.3 | 29.1±4.9 | 27.9±8.3 | 29.4±3.9 | 30.6±4.8 | 27.6±6.0* | 29.8±7.4 | 28.9±4.7 |

EDV: end-diastolic volume; ESV: end-systolic volume; LVEF: ejection fraction; LAV ind; left atrial volume index; MR: mitral regurgitation; E/A: ratio of the early (E) to late (A) ventricular filling velocities; E/e': ratio of the transmitral early peak velocity by pulsed wave Doppler and the early diastolic mitral annulus velocity by tissue Doppler; RVAd: right ventricular end-diastolic area; FAC: fractional area change; TAPSE: tricuspid annular plane systolic excursion; RA area: right atrium area; TR: tricuspid regurgitation; PPS: systolic pulmonary artery pressure. * p<0.01 # p<0.05 § p=0.05 @ p=0.06
